# Dissecting Mismatch Negativity: Early and Late Subcomponents for Detecting Deviants in Local and Global Sequence Regularities

**DOI:** 10.1523/ENEURO.0050-24.2024

**Published:** 2024-05-16

**Authors:** Yiyuan Teresa Huang, Chien-Te Wu, Shinsuke Koike, Zenas C. Chao

**Affiliations:** ^1^International Research Center for Neurointelligence (WPI-IRCN), UTIAS, The University of Tokyo, Tokyo 113-0033, Japan; ^2^School of Occupational Therapy, College of Medicine, National Taiwan University, Taipei 100, Taiwan; ^3^Department of Multidisciplinary Sciences, Graduate School of Arts and Sciences, The University of Tokyo, Tokyo 153-8902, Japan; ^4^University of Tokyo Institute for Diversity & Adaptation of Human Mind (UTIDAHM), Tokyo 113-0033, Japan

**Keywords:** EEG, hierarchy, mismatch negativity, predictive coding, subcomponents

## Abstract

Mismatch negativity (MMN) is commonly recognized as a neural signal of prediction error evoked by deviants from the expected patterns of sensory input. Studies show that MMN diminishes when sequence patterns become more predictable over a longer timescale. This implies that MMN is composed of multiple subcomponents, each responding to different levels of temporal regularities. To probe the hypothesized subcomponents in MMN, we record human electroencephalography during an auditory local–global oddball paradigm where the tone-to-tone transition probability (local regularity) and the overall sequence probability (global regularity) are manipulated to control temporal predictabilities at two hierarchical levels. We find that the size of MMN is correlated with both probabilities and the spatiotemporal structure of MMN can be decomposed into two distinct subcomponents. Both subcomponents appear as negative waveforms, with one peaking early in the central-frontal area and the other late in a more frontal area. With a quantitative predictive coding model, we map the early and late subcomponents to the prediction errors that are tied to local and global regularities, respectively. Our study highlights the hierarchical complexity of MMN and offers an experimental and analytical platform for developing a multitiered neural marker applicable in clinical settings.

## Significance Statement

Our study provides new insights into the intricate architecture of mismatch negativity (MMN), a key neural indicator for deviant detection. Using a refined oddball paradigm with dual-level temporal controls, we identified two unique MMN subcomponents, each linked to prediction errors at different brain hierarchies. This work establishes a practical platform for a multitiered neural marker, offering clinical applications for assessing brain function across various hierarchies.

## Introduction

Mismatch negativity (MMN) is measured by contrasting the event-related potential (ERP) evoked by an infrequent sensory event (referred to as a deviant stimulus) to the ERP from a frequent event (a standard stimulus). This contrast shows a negative waveform peaking at 100–250 ms after event onset and over the frontal-central brain area (Sams et al., 1984; Näätänen et al., 2007). The MMN has been recognized as a key biomarker to evaluate the capacity for statistical learning and deviance detection in various disorders, such as autism spectrum disorder (ASD; Dunn et al., 2008), schizophrenia (Fisher et al., 2012; Erickson et al., 2016; Koshiyama et al., 2020), and developmental delay (Kujala and Näätänen, 2001).

Theoretically, MMN has been interpreted as a form of prediction error within the framework of hierarchical predictive coding theory (Wacongne et al., 2012). The theory postulates that bidirectional cascades operate across the hierarchy to minimize prediction errors: top-down predictions for incoming inputs are generated by learning statistical regularities, and bottom-up prediction errors are generated to refine the predictions when discrepancies between expected and actual sensory inputs occur (Friston, 2010; Bastos et al., 2012; Clark, 2013). The coding has been suggested as an underlying mechanism of the generation of MMN, which has been tested in different oddball paradigms (Garrido et al., 2007, 2008). Furthermore, as the prediction error indirectly reflects the prediction established based on the statistical regularity of stimuli, many studies have also reported that a less frequent stimulus, thus with less predictability, evokes a larger MMN (Javitt et al., 1998; Sato et al., 2000; Sabri and Campbell, 2001).

MMN is also modulated by statistical regularities at a longer timescale. This is evident in a local–global oddball paradigm (Bekinschtein et al., 2009), in which stimulus sequences are manipulated to control temporal regularities at two hierarchical levels: tone-to-tone transitions (local level) and multitone sequence structure (global level). The MMN amplitude triggered by the local deviant is notably smaller as the deviant becomes predictable at the global level (Wacongne et al., 2011), and this reduction is absent in ASD and schizophrenia (Sauer et al., 2017; Goris et al., 2018). These results suggest that different degrees of local and global prediction errors are generated and superimposed in MMN. In the current study, we aim to clearly dissociate the superimposed MMN into subcomponents showing distinct neural signatures. Specifically, we hypothesize that there are two MMN subcomponents, each responding to the local and global levels of temporal statistics and thus representing the local and global prediction-error signals.

We record human electroencephalography (EEG) during an auditory local–global paradigm where stimulus occurrences of the deviant are manipulated at the local and global level to create varying degrees of prediction error in four sequence blocks. To disentangle MMN subcomponents from ERP data, we used an unbiased data-driven decomposition method that was previously applied to isolate prediction-error signals in high-gamma frequency bands (Chao et al., 2018). Two subcomponents are extracted from the data, each exhibiting features of MMN. The first subcomponent is characterized by a negative waveform peaking at 136 ms in the central-frontal area of the brain, and the second subcomponent is also a negative waveform peaking later at 200 ms in a more frontal area. Furthermore, their activation patterns across different sequence blocks are aligned with the predictions from a hierarchical predictive coding model (Chao et al., 2022), suggesting that the early and late MMN subcomponents represent the local and global prediction-error signals, respectively. Our findings reveal the composite nature of MMN and suggest that breaking down MMN into distinct subcomponents may provide a more complete biomarker for pathological conditions associated with unusual prediction mechanisms.

## Materials and Methods

### Participants

Thirty participants were recruited in this study (15 males and 15 females; age, 24 ± 2.6 years old; mean ± standard deviation). The participants self-reported and were screened to have no participation in drug studies and no history of neurological and psychological conditions. This study was approved by the Research Ethics Committee of the National Taiwan University Hospital (201906081 RINA), and all participants gave written informed consent after understanding experimental procedures and before the experiment.

### Local–global oddball paradigm with controlled predictabilities

**Figure 1.**
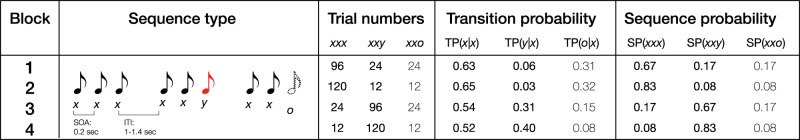
Task design**.** The configuration of sequence types, number of trials, transition probabilities, and sequence probabilities in four blocks. The tone icons colored in black and red represent tone *x* and *y* in different pitches, while the tone icon with the dashed outline represents omission (“*o*”, no tone delivered). The stimulus onset asynchrony (denoted as SOA) represents time interval between the onsets of a tone and the next. The inter-trial interval (ITI) represents time interval between the offset of one sequence's last tone and the onset of the next sequence's first tone. The probabilities were rounded to two decimal places.

We implemented an auditory local–global oddball paradigm with four distinct sequence blocks (Fig. 1). Each block was formed by varying stimulus occurrences for a unique combination of local and global regularities. Two tones were created by combining three sinusoidal waves of base frequencies: the low-pitched tone with 350, 700, and 1,400 Hz and the high-pitched tone with 500, 1,000, and 1,500 Hz. Each tone had a 100 ms duration with a 7 ms rise and fall. Each tone sequence consisted of three tones consecutively delivered with a 200 ms stimulus onset asynchrony (SOA). The intertrial interval (ITI) between the end of one sequence's last tone and the start of the next sequence's first tone was randomly set to a value between 1,000 and 1,400 ms, in 50 ms increments.

For each block, there were three types of tone sequence: *xxx*, representing three tones with the same tone pitch; *xxy*, representing that the last tone differed from the preceding tones; and *xxo*, representing that the last tone was omitted. The numbers of trials for the sequences *xxx*, *xxy*, and *xxo* were set to be 96:24:24 for Block 1, 120:12:12 for Block 2, 24:96:24 for Block 3, and 12:120:12 for Block 4. Each block was sectioned into four phases in which the proportion of sequence types was maintained (e.g., in Block 1, every phase contained 24 trials of *xxx*, 6 trials of *xxy*, and 6 trials of *xxo*). Within each phase, the order of the 36 sequences was random. The four blocks were delivered twice: once with the low-pitched tone as tone *x* and the high-pitched tone as tone *y* and once with the high-pitched tone as tone *x* and the low-pitched tone as tone *y*. The order of eight block presentations was random.

With different sequence ratios, a unique configuration of local and global regularities was created for predictions of the last tone *x* or *y*. On the one hand, the local regularity was established by the tone-to-tone transition probability (TP), i.e., the conditional probability of an incoming tone given the previous tone. TP in each block contained three values, TP(*x*|*x*), TP(*y*|*x*), and TP(*o*|*x*), which represent the conditional probabilities of tones *x*, *y*, and *o* occurring, given the previous tone *x*, within a single block. In sequence *xxx*, there are two transitions from *x* to *x* and one transition from *x* to *o*. In sequence *xxy*, there is one transition from *x* to *x* and one transition from *x* to *y*. In sequence *xxo*, there is one transition from *x* to *x* and one transition from *x* to *o*. Note that the *x*-to-*o* transition was considered at the end of the tone sequence *xxx*, since the sequence ending cannot be known by stimulus transitions but rather by sequence structure (Chao et al., 2022). Then, based on trial numbers of the sequence types, we calculated all instances of the three transition types within one block and the corresponding transition probabilities.

On the other hand, the global regularity was established by the multitone sequence probability (SP). SP in each block contained three values, SP(*xxx*), SP(*xxy*), and SP(*xxo*), which represent the probabilities of sequences *xxx*, *xxy*, and *xxo* within a single block. The four blocks featured distinct combinations of TP and SP (Fig. 1), which allowed us to control the predictability of sensory stimuli and examine varying degrees of prediction error at both local and global levels.

During the experiment, participants were instructed to pay attention to the sound while visually fixating at a central fixation (white cross on a gray background), and no behavioral response was required. A task-irrelevant video was displayed during the break between two block presentations to minimize the influence of the learned regularities in a run being carried over to the next run. All stimulus presentations were programmed with MATLAB-based Psychtoolbox (Pelli, 1997; Kleiner et al., 2007) and presented with a monitor (resolution, 1,920*1,220 pixels; sampling rate, 60 Hz) and a pair of desktop speakers (∼60 dB).

### EEG recording and analysis

Raw EEG data were recorded with a 64-channel QuickCap (Compumedics NeuroScan). During recording, the data were referenced to a reference electrode near Cz, and impedances were kept to <2 kΩ for two mastoid electrodes and 5 kΩ for the remaining electrodes. We set a bandpass filter of 0.01 to 100 Hz and a 500 Hz sampling rate.

We used MATLAB-based EEGLAB (Delorme and Makeig, 2004) to preprocess the data from each participant. First, we merged the data from all runs and rereferenced it to the average of two mastoid electrodes to eliminate systematic noise from the environment (functions: *pop_mergeset.m*; *pop_reref.m*). Second, epochs of sequences were extracted from 1.2 s before the first tone to 1.9 s after the last tone (*pop_epoch.m*). Third, bad epochs containing excessive fluctuations or high-frequency noise were manually removed. On average, ∼2% of the total epochs in each participant were removed at this step. Fourth, we used the ADJUST toolbox to remove eye and muscular artifacts (Mognon et al., 2011; *pop_runica.m*, *interface_ADJ.m*, *pop_subcomp.m*). Finally, the processed epochs were corrected with the baseline estimated within the time window of −300 to −100 ms relative to the onset of the first tone (*pop_rmbase.m*) and downsampled to 250 Hz.

**Figure 2.**
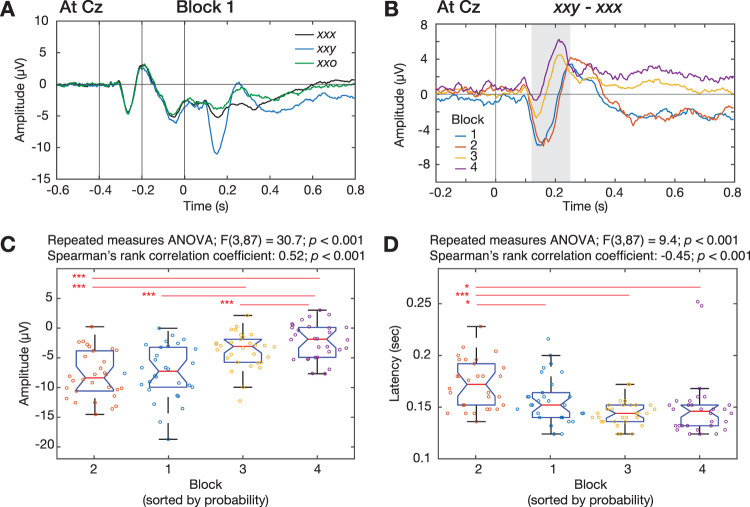
ERPs and deviant responses from contrasts**.** ***A*****,** The group mean ERPs of sequences *xxx*, *xxy*, and *xxo* in Block 1 at channel Cz. Solid vertical lines represent onsets of the stimuli of a sequence. Time zero was set to be the onset of the last stimulus. ***B*****,** Contrast responses obtained by contrasting ERPs of sequences *xxy* and *xxx* in 4 blocks. The gray shade represents the time range of the MMN. ***C*** and ***D*****,** Peak amplitudes and latencies of the MMN at Cz in 4 blocks. The block order was sorted from low to high probability in terms of TP(*y*|*x*) and SP(*xxy*). The 30 dots in each block correspond to participants. The box plot represents the median (red horizontal line), quartiles (the bottom and top edges), 95% confidence interval (notches), and the maximum and minimum (black horizontal lines). Results from repeated measures ANOVA and Spearman's rank correlation analyses are shown above the plots. The red line represents a significant difference between two blocks (*<0.05; ***<0.001).

For each channel, block, and participant, we then averaged the signals across trials to obtain the ERP for each sequence type (i.e., *xxx*, *xxy*, and *xxo*); see examples in Figure 2*A*. To measure the MMN in the local–global oddball paradigm, we compared ERPs from sequence *xxy* (local deviant) to ERPs from sequence *xxx* (local standard) (*xxy* – *xxx*) for each channel, block, and participant. Note that the contrast was done in all conditions including frequent *xxy* as the global standard, consistent with previous studies evaluating varied MMN evoked by the local violation and influenced by different degrees of global expectations (Wacongne et al., 2011; Sauer et al., 2017; Goris et al., 2018). Examples of the contrast responses from −200 to 800 ms relative to the onset of the last tone in the four blocks are shown in Figure 2*B*.

We also estimated the omission error by contrasting ERPs between sequence *xxo* and *xxx* (*xxo* – *xxx*), where positive responses were found ∼100–200 ms (Extended Data Fig. 2-1). However, this contrast response contains the sensory response to the last tone in sequence *xxx* and so does not accurately reflect the true prediction error. Consequently, we did not include the omission trials for further analysis.

10.1523/ENEURO.0050-24.2024.f2-1 Figure 2-1 Download Figure 2-1, TIF file.

### Analysis of the MMN amplitude and latency

We examined effects of the TP (local regularity) and SP (global regularity) on MMN amplitudes and latencies across four blocks using repeated measures ANOVA and Spearman's rank correlation coefficient. To select peak amplitudes and latencies of the MMN for each block and participant, we focused on the minimum of the contrast responses (*xxy* – *xxx*) at Cz in the range of 120–250 ms after the onset of the last tone (Näätänen et al., 2007). Firstly, we implement a repeated measures ANOVA to measure differences in MMN among four blocks, each with unique TP and SP (functions: *fitrm.m* and *ranova.m*). Also, pairwise comparisons with Bonferroni’s correction were implemented for post hoc tests (*multcompare.m*). Secondly, as Sato et al. (2000) showed that MMN amplitudes become larger with fewer occurrences of novel stimuli, we performed Spearman's rank correlation between the MMN and the probability ranked based on TP(*y*|*x*) and SP(*xxy*).

When a stimulus is presented repeatedly, the evoked neural activity is reduced, an effect known as stimulus-specific adaptation. In our study, three identical tones were consecutively presented (*xxx*) or the last was replaced with a different tone (*xxy*). As shown in a previous study (Hofmann-Shen et al., 2020), when comparing the two sequence types, the N1 as an index of the adaptation effect was observed with positive polarity between 80 and 120 ms. We further estimated effects of N1 in the blocks (Extended Data Fig. 2-2). The results of pairwise comparisons showed the size of N1 was not correlated with TP and SP, and this indicates that MMN, but not N1, is varied according to two-level probability manipulations.

10.1523/ENEURO.0050-24.2024.f2-2 Figure 2-2 Download Figure 2-2, TIF file.

**Figure 3.**
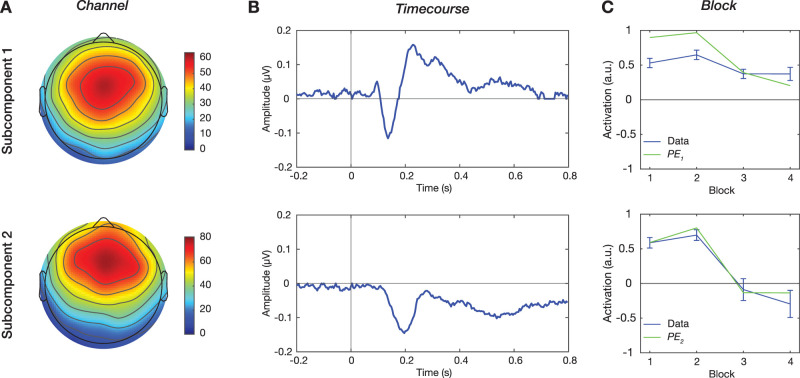
Neural signatures of prediction-error signals**.** We resampled 100 datasets, each containing contrast responses of 4 blocks, and then performed PARAFAC decomposition for each dataset. For the two extracted subcomponents, activations are shown in (***A***) *Channel*, (***B***) *Timecourse*, and (***C***) *Block* dimensions. The *Block* dimension represents activation of the subcomponents to the responses (the blue line). The error bar represents the standard deviation. The model values of the local and global prediction errors are denoted as *PE*_*1*_ and *PE*_*2*_ (the green line).

10.1523/ENEURO.0050-24.2024.f2-3 Figure 2-3 Download Figure 2-3, DOCX file.

### Parallel factor analysis

In our study, the total responses can be pooled in a three-dimensional tensor (62 channels × 250 time points × 4 blocks), which provides a comprehensive description of the spatiotemporal dynamics under different TP and SP combinations. To extract the subcomponents in the tensor that likely overlap in space and time, we used parallel factor analysis (PARAFAC; Harshman and Lundy, 1994) to decompose the data. This approach yields subcomponents within the three corresponding dimensions (Channel × Timecourse × Block), enabling us to quantify their spatiotemporal signatures and distinct activations across blocks.

We resampled the contrast responses of the 30 participants 100 times with the bootstrap method (functions, *datasample.m*). For each resampling, a new tensor was generated from the averaged contrast responses across the resampled participants. For each tensor, we performed PARAFAC analysis using the N-way toolbox (Andersson and Bro, 2000; function, *parafac.m*) to decompose the tensor into multiple subcomponents ranging from 1 to 8, leading to a total of 800 (100*8) decompositions. The convergence criterion was set to be 1 × 10^−6^, and the three dimensions were not constrained in terms of orthogonality or positivity. The optimal number of subcomponents was determined based on the Core Consistency Diagnostic (CORCONDIA; Harshman and Lundy, 1994; Bro and Kiers, 2003). Decomposition with low consistency indicates a poor appropriateness, where high interactions exist between subcomponents. A CORCONDIA of 80–90% is considered a good decomposition and below 50% considered a problematic decomposition (Bro and Kiers, 2003; Pouryazdian et al., 2016).

As the optimal number was two (see details in Results), for all 100 resampled tensors, two subcomponents were extracted and described by activation values (i.e., the score or loading matrix) in their original dimensions of Channel, Timecourse, and Block. The Block dimension represents, for example, how much the contrast response of Block 1 (Block) is contributed by Subcomponent 1 composed of spatial and temporal activation values (Channel and Timecourse). Thus, one subcomponent has four values in the Block dimension, meaning different degrees of contributions from that subcomponent to the contrast responses of the four blocks.

We observed two extracted subcomponents, one with an early negative peak and the other with a late negative peak consistently across 100 resamples. Then we assigned the former one as Subcomponent 1 and the latter one as Subcomponent 2. Examples of subcomponents from 10 resamples are shown in Extended Data Figure 3-1. For Subcomponents 1 and 2, we estimated whether the activation values were significantly different from zero across 100 decompositions in the dimensions of Channel and Timecourse (function, *ft_timelockstatistics.mat*). Note that values in the dimension of Channel were estimated with cluster corrections. Then, average activations are shown with nonsignificant values replaced by zero.

10.1523/ENEURO.0050-24.2024.f3-1 Figure 3-1 Download Figure 3-1, TIF file.

### Functional roles of subcomponents identified by a predictive coding model

To verify the functional roles of the identified subcomponents, we used a predictive coding model which can estimate the size of prediction errors during the local–global oddball paradigm (Chao et al., 2022). The model quantitatively describes prediction and prediction-error signals at two hierarchical levels and has been found to explain predictive coding signaling during the local–global oddball paradigm better than other alternative models.

Using the formula and code provided by Chao et al. (2022), we calculated the theoretical quantities for both the local (or first-level) prediction-error signal and the global (or second-level) prediction-error signal within the deviant-minus-standard difference responses. The local prediction-error signals across the four blocks were represented by four model values (PE_1_), while the global prediction-error signals across the same blocks were characterized by four model values (PE_2_).

Our next step was to examine whether Subcomponents 1 and 2 identified from the data were associated with the local and global prediction-error signals described by the model. To achieve this, we reconstructed deviant responses from Subcomponents 1 and 2 with the model values PE_1_ and PE_2_ and compared them to the actual responses. For each of the 100 decompositions, the response at time *t* for channel *i* and block *j* was reconstructed (denoted as PE1_PE2) as follows:

(1) $$\mathrm{PE}1\_\mathrm{PE}2(i,j,t)=\mathrm{Channe}l_{1}(i)\;*\;PE_{1}(j)\;*\;\mathrm{Timecours}e_{1}(t)+\mathrm{Channe}l_{2}(i)\;*\;PE_{2}(j)\;*\;\mathrm{Timecours}e_{2}(t),$$

where Channel_1_ represents the 62 activation values in the Channel dimension for Subcomponent 1 and Timecourse_1_ represents the 250 activation values in the Timecourse dimension for Subcomponent 1. Similarly, Channel_2_ and Timecourse_2_ represent those values for Subcomponent 2.

We also tested the reconstruction with different associations: Subcomponent 1 as PE_2_ and Subcomponent 2 as PE_1_ (denoted as PE2_PE1), Subcomponents 1 and 2 both as PE_1_ (denoted as PE1_PE1), and Subcomponents 1 and 2 both as PE_2_ (denoted as PE2_PE2).

(2) $$\mathrm{PE}2\_\mathrm{PE}1(i,j,t)=\mathrm{Channe}l_{1}(i)\;*\;PE_{2}(j)\;*\;\mathrm{Timecours}e_{1}(t)+\mathrm{Channe}l_{2}(i)\;*\;PE_{1}(j)\;*\;\mathrm{Timecours}e_{2}(t),$$

(3) $$\mathrm{PE}1\_\mathrm{PE}1(i,j,t)=\mathrm{Channe}l_{1}(i)\;*\;PE_{1}(j)\;*\;\mathrm{Timecours}e_{1}(t)+\mathrm{Channe}l_{2}(i)\;*\;PE_{1}(j)\;*\;\mathrm{Timecours}e_{2}(t),$$

(4) $$\mathrm{PE}2\_\mathrm{PE}2(i,j,t)=\mathrm{Channe}l_{1}(i)\;*\;PE_{2}(j)\;*\;\mathrm{Timecours}e_{1}(t)+\mathrm{Channe}l_{2}(i)\;*\;PE_{2}(j)\;*\;\mathrm{Timecours}e_{2}(t).$$

We then calculated the mean squared difference (MSD) between reconstructed responses and the actual response, Act, for each channel *i* and block *j*. Below, we use the reconstruction of PE1_PE2 as an example:

(5) $$\mathrm{MSD}(i,j)=\frac{1}{250}\overset{250}{\underset{t=1}{\sum }}\;(\mathrm{PE}1_{\mathrm{PE}2}(i,j,t)-\mathrm{Act}(i,j,t){)}^{2}.$$

We also measured Pearson’s correlation coefficients of the reconstructed and actual time courses (*t* = 1:250) for each channel *i* and block *j*:

(6) $$R(i,j)=\mathrm{corr}(PE1_{PE2}(i,j,1:250),\mathrm{Act}(i,j,1:250)).$$

We averaged MSD and *R* across 62 channels and four blocks for each of the 100 decompositions and each of four associations. To evaluate which association produced better reconstruction, average MSD and *R* were tested across the four associations using a repeated measures ANOVA and pairwise comparisons with Bonferroni’s correction.

## Results

### MMN varies across blocks

Figure 2*A* shows an example of ERPs, averaged across participants, from the channel Cz in Block 1. For each block, we extracted MMN by contrasting ERPs between sequences *xxy* and *xxx* (*xxy* – *xxx*). Figure 2*B* shows the average contrast responses from the four blocks, sorted from low TP(*y*|*x*) and SP(*xxy*) to high TP(*y*|*x*) and SP(*xxy*). To examine whether and how the local and global regularities (i.e., TP and SP) affect the peak amplitude and latency of MMN, we performed a repeated measures ANOVA across all blocks and participants. First, we found significant differences in the peak amplitude among the blocks (*F*_(3,87)_ = 30.7; *p *< 0.001; *n* = 30 participants; repeated measures ANOVA; Fig. 2*C*). In post hoc tests (Extended Data Fig. 2-3), the amplitude in Block 1 with fewer occurrences of the deviant *y* was significantly more negative than that in Block 3 (*p *< 0.001; *n* = 30 participants; pairwise comparison; Bonferroni’s correction; Fig. 2*C*, red horizontal line) and Block 4 (*p *< 0.001), both of which consisted of more occurrences of the deviant. Similarly, the amplitude in Block 2 was significantly more negative than that in Block 3 (*p *< 0.001) and Block 4 (*p *< 0.001). There were nonsignificant differences in the rest comparisons between Blocks 1 and 2 and between 3 and 4. Although we did not find the significance when comparing two blocks with close deviant probabilities (e.g., TP of the deviant is 0.03 in Block 2 and 0.06 in Block 1), a trend of the increased TP and SP with the smaller amplitude of the negative peak was observed and further tested (Spearman's rank correlation coefficient: 0.52, *p *< 0.001; *n* = 30 participants).

Second, we also found significant differences in the MMN peak latency among the blocks (*F*_(3,87)_ = 9.4; *p *< 0.001; *n* = 30 participants; repeated measures ANOVA; Fig. 2*D*). In post hoc tests (Extended Data Fig. 2-3), specifically, the latency in Block 2 was significantly greater than that in Block 1 (*p *= 0.01; *n* = 30 participants; pairwise comparison; Bonferroni’s correction), Block 3 (*p *< 0.001), and Block 4 (*p *= 0.01). There were nonsignificant differences in the rest comparisons between each two of Blocks 1, 3, and 4. A negative rank correlation between peak latency and probability was found (Spearman's rank correlation coefficient, −0.45; *p *< 0.001; *n* = 30 participants). Altogether, the contrast responses appeared differently across the blocks; in particular, MMN peaked more negatively and later as TP(*y*|*x*) and SP(*xxy*) became smaller.

### Extracting MMN subcomponents

Upon confirming that the MMN is modulated by TP and SP, we further tested if the MMN contains two overlapping subcomponents that respectively encode TP and SP. We resampled the total data of contrast responses 100 times and used PARAFAC to decompose each of the 100 resampled datasets. Each resampled dataset was decomposed into a designated number of subcomponents, ranging from 1 to 8. The CORCONDIA was quantified to evaluate the consistency of the decomposition. We found an abrupt drop in CORCONDIA, from nearly 100 to 45%, when the decomposition changed from two subcomponents to three subcomponents (*p *< 0.001; *n* = 100 resamples; pairwise comparison; Extended Data Fig. 3-2). This indicates that the optimal number of subcomponents in the deviant responses was two.

10.1523/ENEURO.0050-24.2024.f3-2 Figure 3-2 Download Figure 3-2, TIF file.

The two subcomponents, Subcomponents 1 and 2, were identified across resampled datasets and visualized in the dimensions of space (Channel), time (Timecourse), and function (Block; Fig. 3). Spatially, Subcomponent 1 was found in the central-frontal area, while Subcomponent 2 was found in a more frontal area (Fig. 3*A*). Temporally, both components showed negative waveforms after the last tone but with different latencies (Fig. 3*B*). Subcomponent 1 peaked negatively at 136 ms, followed by a positive response during 200–400 ms. Subcomponent 2 peaked negatively at 200 ms, followed by a long-lasting negative response. Functionally, the two subcomponents had different activation values in the Block dimension (Fig. 3*C*, blue line). For Subcomponent 1, the different positive values in the Block dimension indicates that the spatiotemporal response pattern described by the Channel and Timecourse dimensions appeared in the contrast responses of the four blocks, each with different positive contributions. Interestingly, the contributions of Subcomponent 2 in Blocks 3 and 4, where sequence *xxy* was frequent, were negative. This indicates that Subcomponent 2 contributed to the total MMN in an opposite manner when the local deviant became the global standard.

### Functional roles of the MMN subcomponents

**Figure 4.**
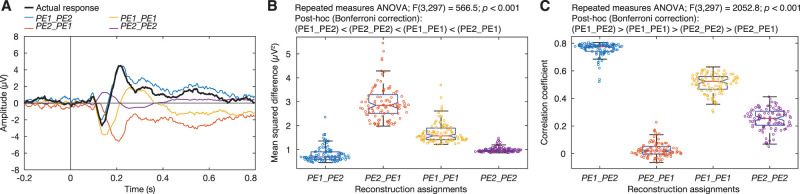
Reconstruction with functional assignments and their differences from the actual ERP**.** ***A*****,** Examples of reconstructed signals at Cz in Block 1. The black line represents the actual contrast response. The blue line represents the reconstructed response with Subcomponent 1 as *PE*_*1*_ and Subcomponent 2 as *PE*_*2*_. The orange line represents the reconstructed response with Subcomponent 1 as *PE*_*2*_ and Subcomponent 2 as *PE*_*1*_. The yellow line represents the reconstructed response with Subcomponent 1 and 2 both as *PE*_*1*._ The purple line represents the reconstructed response with Subcomponent 1 and 2 both as *PE*_*2*._ Mean squared differences in panel ***B*** and Pearson correlation coefficients in panel ***C*** were calculated then averaged across channels and blocks. The 100 dots for each assignment represent resamples*.* Results from repeated measures ANOVA and pairwise comparisons are shown above the plots.

To identify functional roles of Subcomponents 1 and 2, we compared their contributions (Fig. 3*C*, blue line) across the four blocks (Block_1_ and Block_2_) to the theoretical values of the local and global prediction errors (PE_1_ and PE_2_) obtained from a hierarchical predictive coding model (Fig. 3*C*, green line). Based on their spatiotemporal characteristics, we hypothesized that Subcomponent 1, occurring earlier in a less frontal area, represented the local prediction error. On the other hand, Subcomponent 2, occurring later in a more frontal area, was hypothesized to represent the global prediction error. To validate the hypothesis, we reconstructed signals with the model values and compared them to actual contrast responses. Figure 4*A* shows examples of reconstructed time courses at channel Cz in Block 1. In this example, the reconstruction PE1_PE2, in which Subcomponent 1 is associated with PE_1_ and Subcomponent 2 with PE_2_, was very similar to the actual response. Among the four associations, the mean square error evaluated with the association of PE1_PE2 was significantly smaller than the other three (all *p *< 0.001; *n* = 100 resamples; pairwise comparisons; Bonferroni’s correction; Fig. 4*B*). Similarly, the correlation coefficient evaluated with the association of PE1_PE2 was significantly higher than the other three (all *p *< 0.001; Fig. 4*C*). These results suggest that Subcomponents 1 and 2 represented the local and global prediction-error signals, respectively.

## Discussion

In the current study, we combined an extended auditory local–global oddball paradigm with a data-driven decomposition analysis to extract subcomponents in MMN. We further demonstrated that the early MMN subcomponent in the central-frontal area represents the local prediction-error signal while the late MMN subcomponent in the more frontal area represents the global prediction-error signal. Our study applies the predictive coding framework to reveal the complex nature of MMN and establishes a robust experimental and analytical platform that can examine the functionality of multilevel deviant detection in both healthy and affected brains.

### Hierarchical MMN in the predictive coding framework

We revealed that the MMN of the deviant response varied with the hierarchical probabilities of stimulus presentation and notably consisted of two MMN subcomponents with distinct spatial and temporal characteristics obtained from an unbiased decomposition.

The two subcomponents spatially comply with the results of the hierarchical network which is a three-level hierarchy of cortical cascades in response to auditory changes. The network consists of bilateral primary auditory cortices receiving inputs, superior temporal gyrus functioning as a memory trace of priors, and right inferior frontal gyrus modulating the attention allocation (Giard et al., 1990; Opitz et al., 2002; Doeller et al., 2003; Halgren et al., 2011; MacLean and Ward, 2014; Hofmann-Shen et al., 2020). It has also been applied to model data obtained in different types of oddball paradigms (Garrido et al., 2007, 2008). In our findings, the early MMN was activated in the frontal-central area, which is consistent with previous literature and may reflect dipole sources in the superior temporal area (Alho et al., 1998; Molholm et al., 2005; Schönwiesner et al., 2007). On the other hand, the late MMN was activated in the more frontal area and slightly dispersed toward the right side, which may be mapped to an anterior portion of the brain such as the right prefrontal cortex. Although EEG signals are limited in spatial resolution, the temporal patterns of the EEG signals may provide some hints regarding potential sources. Specifically, we found that the early subcomponent (i.e., Subcomponent 1) of MMN at the central-frontal area was elicited earlier and was followed by the late subcomponent (Subcomponent 2) of MMN in the frontal area, which is consistent with differences in the peak latency found in the temporal and frontal generations of MMN (Rinne et al., 2000) and bottom-up gamma oscillations (Chao et al., 2022). Furthermore, in alignment with the hierarchical predictive coding theory, the observed temporal pattern supported feedforward error propagating between low and high cortical hierarchy, and activations of MMN subcomponents closely resembled the quantitative definition of local and global prediction-error signals (Chao et al., 2022).

However, the findings and interpretation of the early and late subcomponents of MMN appear to deviate from previous studies of the local–global oddball paradigm. It has been claimed that the MMN represents the local prediction-error signal while the P300 represents the global prediction-error signal (Wacongne et al., 2011). This stands in our contrast with our finding of MMN encoding both local and global prediction errors. We argue that this discrepancy results from the difficulty of dissociating hierarchical prediction-error signals. P300 in previous literature was typically captured by contrasting responses to the global deviant and the global standard. In fact, the local prediction-error signal cannot be fully controlled in this contrast. For example, two tones are both defined as the global deviant, while one is also defined as the local deviant and the other is defined as the local standard. When responses of theses tones are combined, different transition probabilities for the tones lead to different local predictability. Therefore, the local prediction is difficult to fully cancel out and still plays a part in the contrast product. To separate subcomponents from ERP contrasts, our approach adopted a decomposition approach. The two extracted subcomponents with quantitative values of hierarchical prediction errors then led to reconstructions similar to the actual responses. The reduction of the MMN when the deviant became more predictable on the global level (i.e., Block 3 and Block 4) can also be explained by Subcomponent 2 with negative contributions in these two blocks. Moreover, in Subcomponent 2, the late MMN peaking at ∼200 ms shared similarities with the N2b component, a negative ERP in the N2 family occurring ∼200–300 ms and after MMN (also known as N2a; Näätänen and Picton, 1986; Pritchard et al., 1991; Folstein and Van Petten, 2008). It has been studied in domains of attention, response inhibition, and cognitive control, where participants are required to pay active attention or make behavioral responses (Kasai et al., 1999; Barry and De Blasio, 2015). This relatively late-stage process coincides with cognitive demands for attentional control and awareness in learning the global regularity in the local–global task (Bekinschtein et al., 2009).

### Limitations and future research

Although our findings provide a more thorough understanding of hierarchical MMN, there are still some limitations to be resolved and advances to be achieved in future research: hierarchical structure composed of more than two levels, prediction updates over time, and neuronal mechanism of learning statistical regularities at two levels.

Firstly, the functional hierarchy underlying MMN is not limited to two levels, as the prediction error likely does not propagate only across two levels of the hierarchical scheme. For example, the encoding of natural images was modeled from the predictive coding perspective, and multiple layers were established to simulate visual cortical processing (Rao and Ballard, 1999; Spratling, 2017). Temporal sequences can consist of not only single-stimulus transition rules but also arbitrary regularities (Dehaene et al., 2015). In order to extract multilevel prediction-error signals in a real sequential environment, probability calculations for different regularities and quantitative values of the prediction error at each level should be determined.

Secondly, the prediction error is generated to update the precision of the prediction; that is, a prediction update follows a prediction error evoked by sensory input. In this study, we observed that the global prediction-error signals had a long-lasting negative waveform after MMN, which might be related to desynchronized beta oscillations as prediction updates (Bastos et al., 2012; Chao et al., 2018). However, the current method is incapable of dissociating the prediction update and the prediction error because of their interdependence; that is, how much the prediction error is generated leads to how much the prediction will be updated. Also, the signal quality in human EEG makes tracking the dynamics of prediction updates during learning challenging. Future research could incorporate analytical techniques with a focus on temporal order to dissociate the prediction update from the prediction error and trial-by-trial analysis to capture the dynamics.

Thirdly, for the oddball paradigm with complex sequence structure, the underlying mechanism of how multiple regularities interact is computed at the neuronal level in the proposed quantitative predictive coding model. The links between the neuronal computations of the regularities and the MMN subcomponents are critically needed for further investigation. We should consider a combination of a biologically realistic model and a microscopical approach such as calcium imaging and high-density neural recording in the future.

## Data Availability

We shared raw EEG data, processed EEG data, and a script for model values of the quantitative predictive coding model on Open Science Framework. Huang, Y. T., Wu, C.-T., Koike, S., & Chao, Z. C. (2024). Dissecting Mismatch Negativity: Early and Late Subcomponents for Detecting Deviants in Local and Global Sequence Regularities. https://doi.org/10.17605/OSF.IO/8YFT4. The raw data featured in this work was previously published in Chao et al. (2022). In the previous work, we constructed a theoretical model specifically designed to quantify hierarchical prediction and prediction-error signals, without placing an emphasis on ERP and MMN.

## Synthesis

Reviewing Editor: Christine Portfors, Washington State University Decisions are customarily a result of the Reviewing Editor and the peer reviewers coming together and discussing their recommendations until a consensus is reached. When revisions are invited, a fact-based synthesis statement explaining their decision and outlining what is needed to prepare a revision will be listed below. The following reviewer(s) agreed to reveal their identity: Jinxing Wei. Note: If this manuscript was transferred from JNeurosci and a decision was made to accept the manuscript without peer review, a brief statement to this effect will instead be what is listed below.

This manuscript provides new insights into the intricate architecture of mismatch negativity (MMN), a key neural indicator for deviant detection. Using a refined oddball paradigm with dual-level temporal controls, they identified two unique MMN subcomponents, each linked to prediction errors at different brain hierarchies. This work establishes a practical platform for a multi-tiered neural marker, offering clinical applications for assessing brain function across various hierarchies.

The reviewers have only have some minor comments.

(1). In the introduction part, the author should explicitly state the specific aim or objective of your study at the end of the introduction. This will provide readers with a clear understanding of what you intend to achieve with your research.

(2). Additionally, while you briefly touch upon the theoretical framework of hierarchical predictive coding theory, consider providing a bit more context or explanation for readers who may not be familiar with this theory. This could involve defining key terms or concepts and explaining how they relate to MMN.

(3). In the line 5, where is the block 5 in figure 2A?

(4). The description for figures 2C and 2D lacks clarity and should provide more details regarding the statistical methods employed. Specifically, the author should elaborate on the statistical tests utilized to analyze the data presented in these figures.

(5). Regarding figure 2C, it is unclear whether there is a difference between block 1 and block 2. The author should explicitly state whether any statistical analysis was conducted to compare the data between these blocks. If there is a difference, the nature of this difference should be described in detail. Similarly, in figure 2D, it is unclear whether there is a difference between block 3 and block 4. The author should provide clarification on whether statistical tests were performed to compare the data between these blocks. Additionally, if a difference exists, the author should explain the implications of this difference.

## Author Response

Synthesis Statement for Author (Required):

This manuscript provides new insights into the intricate architecture of mismatch negativity (MMN), a key neural indicator for deviant detection. Using a refined oddball paradigm with dual-level temporal controls, they identified two unique MMN subcomponents, each linked to prediction errors at different brain hierarchies. This work establishes a practical platform for a multi-tiered neural marker, offering clinical applications for assessing brain function across various hierarchies.

We thank the reviewers for understanding the key points of our paper.

The reviewers have only some minor comments. (1). In the introduction part, the author should explicitly state the specific aim or objective of your study at the end of the introduction. This will provide readers with a clear understanding of what you intend to achieve with your research.

We emphasize our specific research aim in Introduction section: "In the current study, we aim to clearly dissociate the superimposed MMN into subcomponents showing distinct neural signatures. Specifically, we hypothesize that there are two MMN subcomponents, each responding to the local and global levels of temporal statistics and thus representing the local and global prediction-error signals." (page 6, lines 8-12) (2). Additionally, while you briefly touch upon the theoretical framework of hierarchical predictive coding theory, consider providing a bit more context or explanation for readers who may not be familiar with this theory. This could involve defining key terms or concepts and explaining how they relate to MMN.

We further emphasize the cores of the theory: two bi-directional signaling, hierarchical structure, and prediction-error minimization. In addition, we add recent evidence showing that MMN evoked in different oddball paradigms can be explained by computational models based on the predictive coding principle. We also add that the prediction error can be influenced by the statistical regularity, indicated by studies showing MMN changing with the probability manipulation.

We revise and add the details in the second paragraph of Introduction section: "The theory postulates that bi-directional cascades operate across the hierarchy to minimize prediction errors: top-down predictions for incoming inputs are generated by learning statistical regularities, and bottom-up prediction errors are generated to refine the predictions when discrepancies between expected and actual sensory inputs occur (Bastos et al., 2012a; Clark, 2013; Friston, 2010). The coding has been suggested as an undelaying mechanism of the generation of MMN, which has been tested in different oddball paradigms (Garrido et al., 2007, 2008). Furthermore, as the prediction error indirectly reflects the prediction established based on the statistical regularity of stimuli, many studies have also reported that a less frequent stimulus, thus with less predictability, evokes a larger MMN (Javitt et al., 1998; Sabri & Campbell, 2001;

Sato et al., 2000)." (page 5, lines 12-21) (3). In the line 5, where is the block 5 in figure 2A? This is a misspelling, and we delete it. (4). The description for figures 2C and 2D lacks clarity and should provide more details regarding the statistical methods employed. Specifically, the author should elaborate on the statistical tests utilized to analyze the data presented in these figures.

We add details of statistical tests in Result section. "..., we performed a repeated measures ANOVA across all blocks and participants. First, we found significant differences in the peak amplitude among the blocks (F[3,87] = 30.7, p < 0.001, n = 30 participants, repeated measures ANOVA, Figure 2C)." (page 18, lines 8-11) "... (p < 0.001, n = 30 participants, pairwise comparison, Bonferroni correction, Figure 2C, the red horizontal line)..." (page18, lines 13-14) "... (Spearman's rank correlation coefficient: 0.52, p < 0.001, n = 30 participants)." (page18, lines 20-21) "... (F[3,87] = 9.4, p < 0.001, n = 30 participants, repeated measures ANOVA, Figure 2D)." (page19, line 2) "...(p = 0.01, n = 30 participants, pairwise comparison, Bonferroni correction)..." (page19, line 4) "...(Spearman's rank correlation coefficient: -0.45, p < 0.001, n = 30 participants)." (page19, lines 7-8) We add statistical methods in Figures 2C and 2D, Figures 4B and 4C, and Figure 2-2. (5). Regarding figure 2C, it is unclear whether there is a difference between block 1 and block 2.

The author should explicitly state whether any statistical analysis was conducted to compare the data between these blocks. If there is a difference, the nature of this difference should be described in detail. Similarly, in figure 2D, it is unclear whether there is a difference between block 3 and block 4. The author should provide clarification on whether statistical tests were performed to compare the data between these blocks. Additionally, if a difference exists, the author should explain the implications of this difference.

We address this by adding labels for significant pairwise comparisons in Figures 2C and 2D, and summarizing pairwise comparison results in Figure 2-3. I will explain this in more detail below.

- In Figure 2C, which displays the differences in the peak amplitude among four blocks, there is no significant difference between Block 1 and Block 2 as well as between Block 3 and 4. The statistics were obtained using pairwise comparisons with Bonferroni correction, after the significant difference among the four blocks was obtained using repeated measures ANOVA.

Figure 2-3 shows results of the pairwise comparisons (below). The statistic for the non- significant difference between Block 1 and Block 2 was highlighted in yellow. We observed non-significant differences between two blocks with close deviant probabilities (e.g., TP of the deviant is 0.03 in Block 2 and 0.06 in Block 1), but a trend of increased deviant probabilities with smaller MMN size, which was also tested using Spearman's rank correlations.

- In Figure 2D, which displays the differences in the peak latency among four blocks, there is no significant difference between Block 3 and Block 4 as well as between Blocks 1 and 3 and between Blocks 1 and 4. The statistics were obtained as the above-mentioned methods and shown in the table. We observed non-significant differences among Blocks 1, 3, and 4, but a trend of increased deviant probabilities with earlier MMN onset, which was also tested using Spearman's rank correlations.

We add detailed results in Result section. "In post-hoc tests (Figure 2-3), ..." (page 18, lines 11-12; page 19, lines 2-3) "There were non-significant differences in the rest comparisons between Blocks 1 and 2 and between 3 and 4. Although we did not find the significance when comparing two blocks with close deviant probabilities (e.g., TP of the deviant is 0.03 in Block 2 and 0.06 in Block 1), a trend of the increased TP and SP with the smaller amplitude of the negative peak was observed and further tested (Spearman's rank correlation coefficient: 0.52, p < 0.001, n = 30 participants)." (page 18, lines 16-21) "There were non-significant differences in the rest comparisons between each two of Blocks 1, 3, and 4." (page 19, lines 5-6)
