# Supplementary figures and images for: Dissecting Mismatch Negativity: Early and Late Subcomponents for Detecting Deviants in Local and Global Sequence Regularities
Source: eNeuro. 2024 May 16;11(5):ENEURO.0050-24.2024. doi: 10.1523/ENEURO.0050-24.2024 (PMC11103647; doi:10.1523/ENEURO.0050-24.2024)

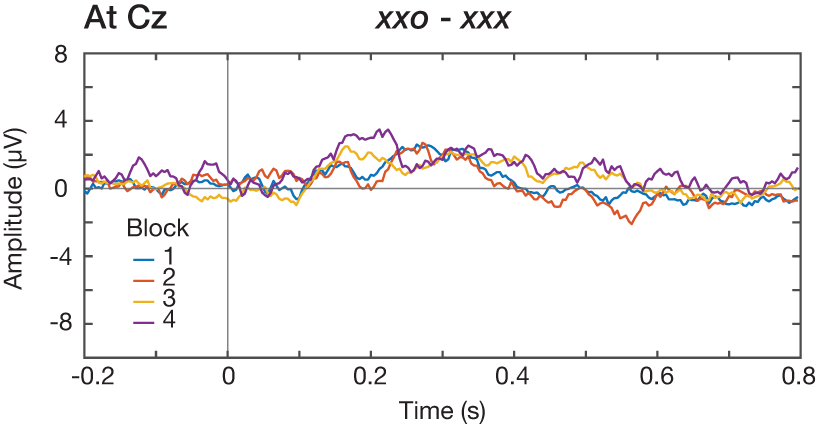

Supplement: Figure 2-1 — Download Figure 2-1, TIF file. [file eneuro-11-ENEURO.0050-24.2024-s001.tif]

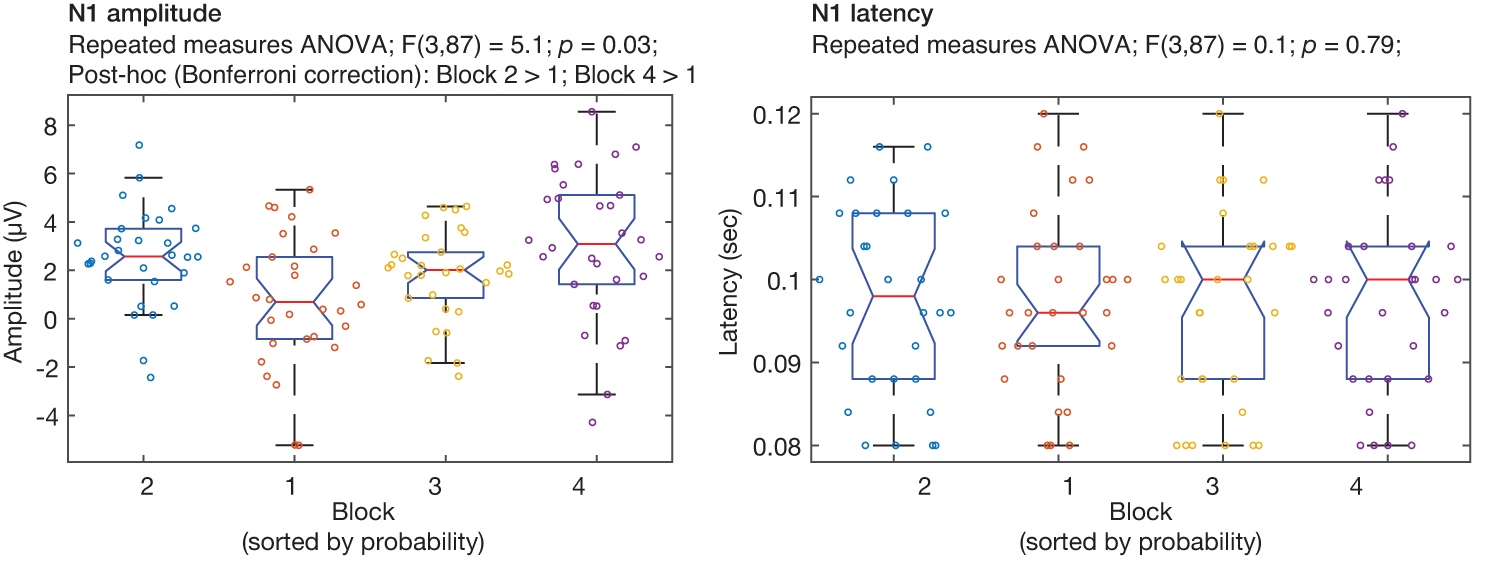

Supplement: Figure 2-2 — Download Figure 2-2, TIF file. [file eneuro-11-ENEURO.0050-24.2024-s002.tif]

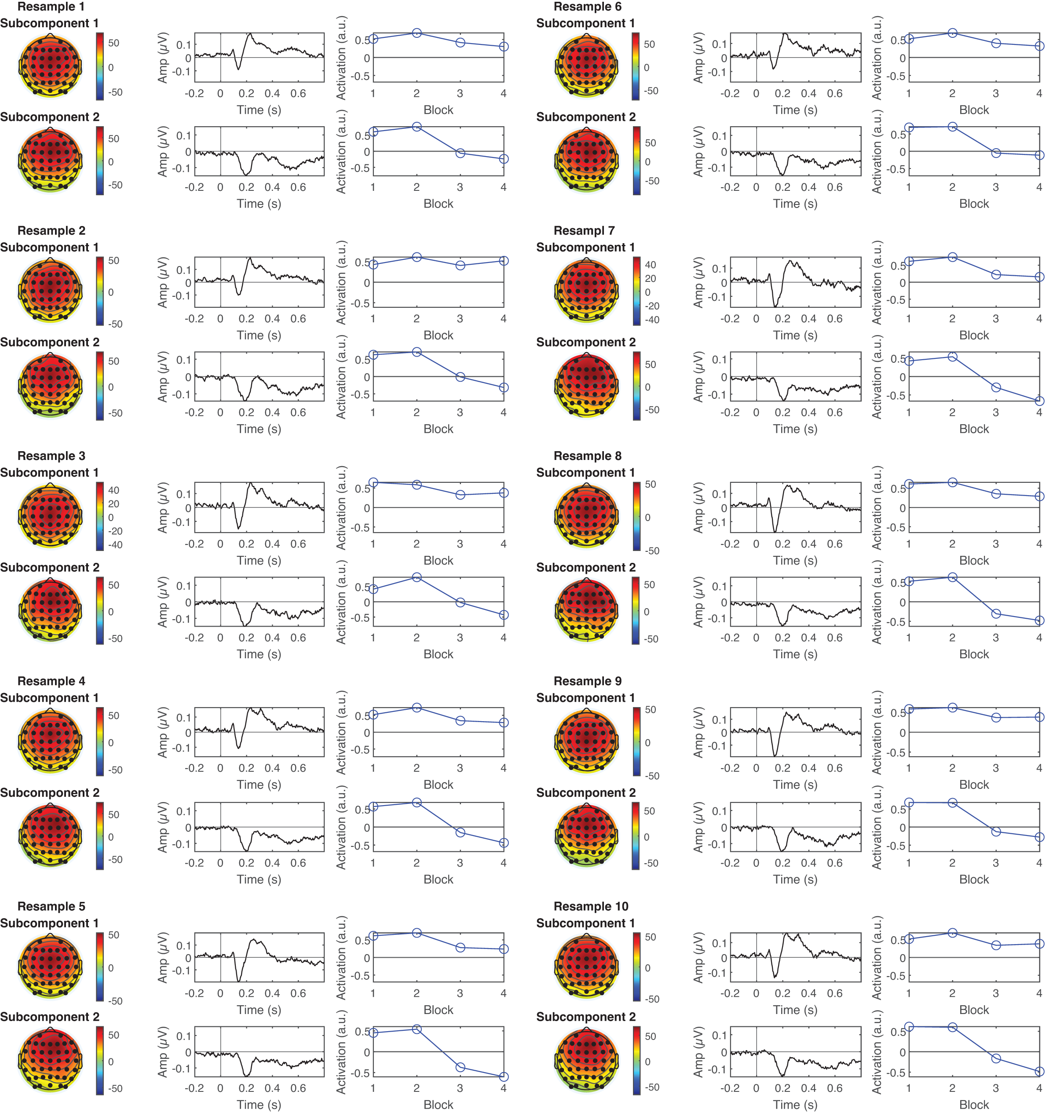

Supplement: Figure 3-1 — Download Figure 3-1, TIF file. [file eneuro-11-ENEURO.0050-24.2024-s004.tif]

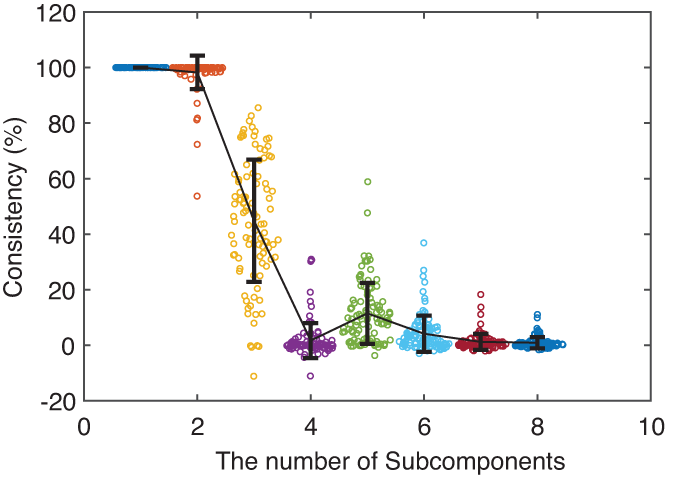

Supplement: Figure 3-2 — Download Figure 3-2, TIF file. [file eneuro-11-ENEURO.0050-24.2024-s005.tif]
