# Supplementary material for: Dissecting Mismatch Negativity: Early and Late Subcomponents for Detecting Deviants in Local and Global Sequence Regularities
Source: eNeuro. 2024 May 16;11(5):ENEURO.0050-24.2024. doi: 10.1523/ENEURO.0050-24.2024 (PMC11103647; doi:10.1523/ENEURO.0050-24.2024)
Supplement: Figure 2-3 — Download Figure 2-3, DOCX file. [file eneuro-11-ENEURO.0050-24.2024-s003.docx]

**Figure 2-3**

**Post-hoc pairwise comparisons**

|  | Peak amplitude  (micro voltage) | | Peak latency  (second) | |
| --- | --- | --- | --- | --- |
|  | Difference  (mean $\pm$ std) | *p* | Difference  (mean $\pm$ std) | *p* |
| Block 2 － Block 1 | -0.51 $\pm$ 0.65 | 1 | 0.016 $\pm$ 0.005 | 0.016 |
| Block 2 － Block 3 | -4.18 $\pm$ 0.62 | <0.001 | 0.028 $\pm$ 0.004 | <0.001 |
| Block 2 － Block 4 | -5.76 $\pm$ 0.87 | <0.001 | 0.023 $\pm$ 0.007 | 0.015 |
| Block 1 － Block 3 | -3.67 $\pm$ 0.61 | <0.001 | 0.013 $\pm$ 0.005 | 0.073 |
| Block 1 － Block 4 | -5.25 $\pm$ 0.80 | <0.001 | 0.007 $\pm$ 0.007 | 1 |
| Block 3 － Block 4 | -1.58 $\pm$ 0.70 | 0.19 | -0.005 $\pm$ 0.005 | 1 |
